# P2Y13 Receptor Regulates HDL Metabolism and Atherosclerosis *In Vivo*

**DOI:** 10.1371/journal.pone.0095807

**Published:** 2014-04-25

**Authors:** Marine Goffinet, Claudine Tardy, Nadia Boubekeur, Guy Cholez, Alice Bluteau, Daniela C. Oniciu, Narendra D. Lalwani, Jean-Louis H. Dasseux, Ronald Barbaras, Rudi Baron

**Affiliations:** 1 Cerenis Therapeutics SA, Labege, France; 2 Cerenis Therapeutics Inc., Ann Arbor, Michigan, United States of America; Harvard Medical School, United States of America

## Abstract

High-density lipoprotein (HDL) is known to protect against atherosclerosis by promoting the reverse cholesterol transport. A new pathway for the regulation of HDL-cholesterol (HDL-c) removal involving F1-ATPase and P2Y13 receptor (P2Y13R) was described *in vitro*, and recently in mice. However, the physiological role of F1-ATPase/P2Y13R pathway in the modulation of vascular pathology i.e. in the development of atherosclerotic plaques is still unknown. We designed a specific novel agonist (CT1007900) of the P2Y13R that caused stimulation of bile acid secretion associated with an increased uptake of HDL-c in the liver after single dosing in mice. Repeated dose administration in mice, for 2 weeks, stimulated the apoA-I synthesis and formation of small HDL particles. Plasma samples from the agonist-treated mice had high efflux capacity for mobilization of cholesterol *in vitro* compared to placebo group. In apoE^−/−^ mice this agonist induced a decrease of atherosclerotic plaques in aortas and carotids. The specificity of P2Y13R pathway in those mice was assessed using adenovirus encoding P2Y13R-shRNA. These results demonstrate that P2Y13R plays a pivotal role in the HDL metabolism and could also be a useful therapeutic agent to decrease atherosclerosis. In this study, the up-regulation of HDL-c metabolism via activation of the P2Y13R using agonists could promote reverse cholesterol transport and promote inhibition of atherosclerosis progression in mice.

## Introduction

The protective effect of high density lipoprotein (HDL) against atherosclerosis is in large part attributed to its ability to mobilize cholesterol from the vessel wall, in particular from lipid-rich atherosclerotic plaques, and transport it to the liver where it is excreted from the body in the form of bile acids or free cholesterol, in a process called reverse cholesterol transport [1], [2]. HDL is also believed to protect against atherosclerosis potentially through anti-inflammatory, antithrombotic, anti-oxidative mechanisms and nitric oxide effects [3]. Multiple mechanisms of action, as well as, limited ability to measure these properties, make HDL a complex therapeutic target albeit with a tremendous potential for the treatment of patients with atherosclerosis. There have been several attempts to increase the HDL-cholesterol (HDL-c) levels using pharmacological intervention. HDL-c levels have been reported to be increased upon chronic administration of fibrates in animals and also in humans [4]. Niacin is the only drug that has been available for patients to raise HDL-c [5]. However, recent data on a large clinical trial with niacin did not show any significant improvement in the cardiovascular risk over statins, commonly used drugs for lipid management. Recently a few attempts have been made to inhibit cholesterol ester transfer protein (CETP) to raise the levels of HDL-c [6]. However, CETP inhibitors continue to face safety hurdle and have not resulted in any significant clinical benefit with these pharmacological regimens [6]. A recent report by Voight et al [7] described that the sole increase in the HDL-c in human, will not necessarily improve the myocardial infarction [7]. The limitations in our ability to predict cardiovascular risk using HDL-c and even Low-density lipoprotein cholesterol (LDL-c) have fueled efforts to identify novel risk markers and to refine the measurement of traditional risk factors, such as LDL-c and HDL-c. Standard assays to evaluate LDL-c and HDL-c quantify the cholesterol content within the respective lipoprotein fraction which only indicate the distribution of cholesterol in different lipoprotein particles at a given time. However, these measures do not indicate if the lipoprotein particles are functional and actively transferring cholesterol for elimination. Both LDL particles (LDL-p) and HDL particles (HDL-p) vary in their content of cholesterol, and thus determining the concentration of lipoprotein particles may be more predictive rather than estimating the cholesterol concentration in assessing the cardiovascular risk. This hypothesis was supported by the data from the Framingham offspring study on LDL-c [8] and more recently for HDL-c in the MESA (Multi-Ethnic Study of Atherosclerosis) trial [9]. This report demonstrated a more consistent inverse association between cardiovascular endpoints and HDL-p compared with HDL-c. These findings suggest that a direct quantification of the concentration of HDL-p may be more useful to define the cardiovascular risk and to evaluate novel HDL-directed therapies which could modulate such lipoprotein particles metabolism [10].

The recent discovery of a new pathway in liver involving F1-ATPase and P2Y13 receptor (P2Y13R) [11] that regulates HDL-cholesterol (HDL-c) removal has given insight and improved our understanding of the HDL metabolism [12]. Indeed, it is believed that the presence of a nucleotidase activity of F1-ATPase subunit at the cell surface of hepatocytes, allows the hydrolysis of ATP to ADP which in turn stimulates the P2Y13R resulting in the uptake of the HDL-c by the cells [13]. More recently, Fabre et al [14] confirmed the relationship between P2Y13R and the reverse cholesterol transport in mice. However, the contribution of the F1-ATPase/P2Y13R pathway in the modulation of HDL function and in the atherosclerotic pathology is not clearly understood [15].

In the present study we demonstrated that the activation of P2Y13R using an agonist ligand results in enhancement of the reverse cholesterol transport. The increase in HDL metabolism subsequently results in the prevention of the progression of atherosclerosis in the animal models suggesting potential therapeutic utility of P2Y13R agonists in the treatment and prevention of atherosclerosis-related disorders.

## Results

In this study, we used new heterocyclic compounds that were designed as orally active P2Y13R agonists (see Figure S1 for structure). These compounds were selected based on their specific activity in 1321N1 cells expressing P2Y13 receptors [16]. Due, to the existence of DNA sequence homologies (around 70%) between P2Y13R and P2Y12R (highly expressed in platelets [17]), the chemical series was also evaluated for its ability to inhibit or stimulate the ADP dependent P2Y12R activity by measuring platelets aggregation. No agonist or antagonist activity on platelet aggregation was observed at a concentration of 30 µM (data not shown). They were further tested for their propensity to stimulate [^3^H]-cholesterol-ether-labelled-HDL uptake *in vitro* on human hepatoma cells (HepG2).

**Figure 1:**
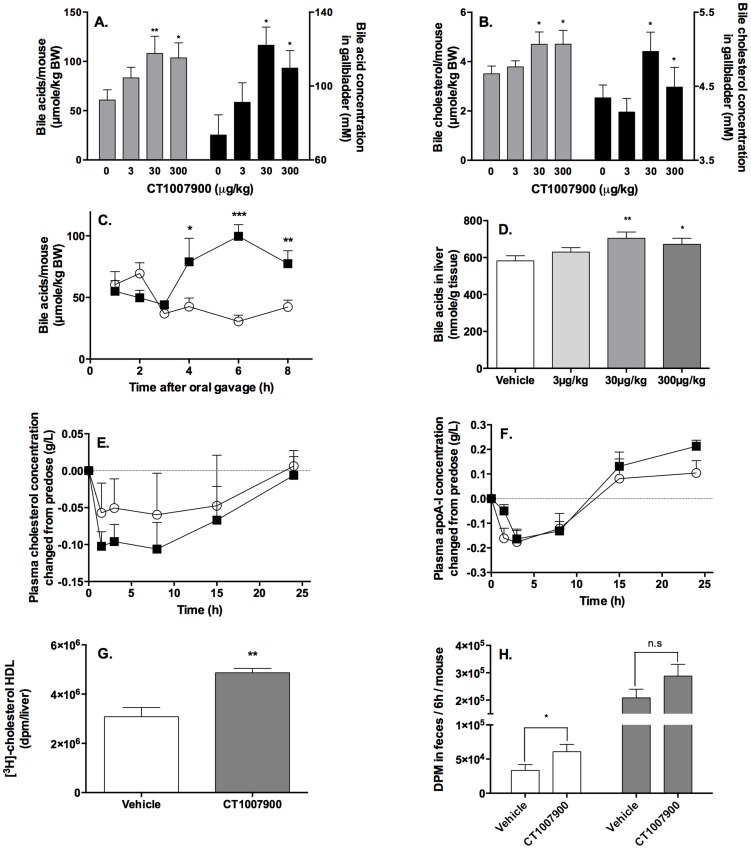
Increase of HDL recycling following activation of P2Y13R pathway in mice. C57Bl/6J mice (n = 10) were fasted for 2 h followed by single oral dose of P2Y13R agonist CT1007900 at 3, 30 or 300 µg/kg. Six hours later bile acid content (panel A) and bile cholesterol content (panel B) were evaluated using enzymatic kits. Grey bars represent the amount of bile acid or bile cholesterol per mouse; black bars represent the concentrations of bile acid and bile cholesterol into gallbladder. Panel C, the kinetic of bile acid mobilization in gallbladder induced by CT1007900 at 300 µg/kg (???) by oral gavage (single dosing) using C57Bl/6J mice (n = 5) was evaluated and compared to vehicle treated animals (○). *p<0.05, **p<0.01, ***p<0.0005. Bile acid content of liver (panel D) was evaluated using enzymatic kit. * p<0.05, **p<0.01. Plasma cholesterol (panel E) and plasma apoA-I (panel F) concentrations were determined at different time points after single oral dose of P2Y13R agonist at 100 µg/kg (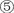) and compared to vehicle treated animals (○). Values in pre-dose groups for plasma cholesterol vary from 0.85 to 0.95 g/L, and 1.1 to 1.3 g/L for plasma apoA-I. Panel G, C57Bl/6J mice (n = 5) were intravenously injected with [^3^H]-cholesterol-labelled mouse HDL (10 µCi/mouse) and CT1007900 (10 nmole/kg or 4 µg/kg). Radioactivity present in the liver was determined 2 hours later. **p<0.01. Panel H, C57Bl/6J mice (n = 5) were dosed (single dosing) with CT1007900 (100 µg/kg) and intravenously injected with [^3^H]-cholesterol-labelled mouse HDL (10 µCi/mouse). Feces from individual mouse were collected for 6 h and extracted for cholesterol (empty bars) and bile acid content (grey bars) and the radioactivity was determined by scintillation counting. *p<0.05.

Since P2Y13R is highly expressed in liver as compared to other tissues [18], [19], we hypothesized that administration of P2Y13R agonists in animals would increase the reverse cholesterol transport, i.e. the HDL-c uptake by the liver and subsequently the bile acid and bile cholesterol secretions. To evaluate this hypothesis, when C57Bl/6J mice were given P2Y13R agonist, CT1007900, by oral gavage, we observed an increase in bile acid and bile cholesterol concentrations into the galbbladder in a dose dependent manner at doses as low as 30 µg/kg (Figure 1A and 1B – black bars). The bile acid and bile cholesterol secretions are stimulated as we reported more bile acid and bile cholesterol per mouse (Figure 1A and 1B – grey bars). Change in bile acid secretion is observed 4 h after oral dosing with CT1007900 and reached a maximum in about 6 h at 300 µg/kg (Figure 1C). Most of the metabolised cholesterol daily is in the form of bile acids and under the dependence of Cholesterol-7α-hydroxylase (CYP7A1) [20]. In mice 75% of the bile acid pool is regulated by CYP7A1 [21]–[23]. The increase in bile acid secretion observed with P2Y13R agonists could be a consequence of either a secretion of bile acids from an intra-hepatic pool to the bile duct [24] or a *de novo* synthesis of bile acids by the liver following stimulation of the HDL-c uptake by the P2Y13R pathway. An increase in liver bile acid content at 30 µg/kg of P2Y13R agonist (Figure 1D) as well as an increase of CYP7A1 mRNA expression (1.7±0.4 fold, p<0.05, 1 h post oral gavage – data not shown) was clearly demonstrated which is in agreement with the stimulation of HDL-c uptake by the liver that results in an increase in the bile acid synthesis. As expected, the plasma cholesterol was significantly decreased (Figure 1E), however, apoA-I levels were not affected compared to the control group (Figure 1F).

To further delineate the implication of the HDL in the increase of bile secretion after treatment of the mice with CT1007900, we performed *in vivo* liver uptake analysis of [^3^H]-cholesterol-labelled mouse HDL (Figure 1G). The intravenous treatment of the animals at 10 nmole/kg with the P2Y13R agonist showed a specific stimulation of the uptake of the [^3^H]-labelled-HDL-c as well as an increased excretion of both [^3^H]-labelled-bile acid and -cholesterol in the feces (Figure 1H) which supports the hypothesis of a stimulation of the HDL-mediated reverse cholesterol transport.

Altogether, these data demonstrated that the stimulation of the P2Y13R pathway resulted in an increase of the hepatic HDL uptake, which in turn increased bile acid and bile cholesterol secretions, and elimination of cholesterol into the feces.

**Figure 2:**
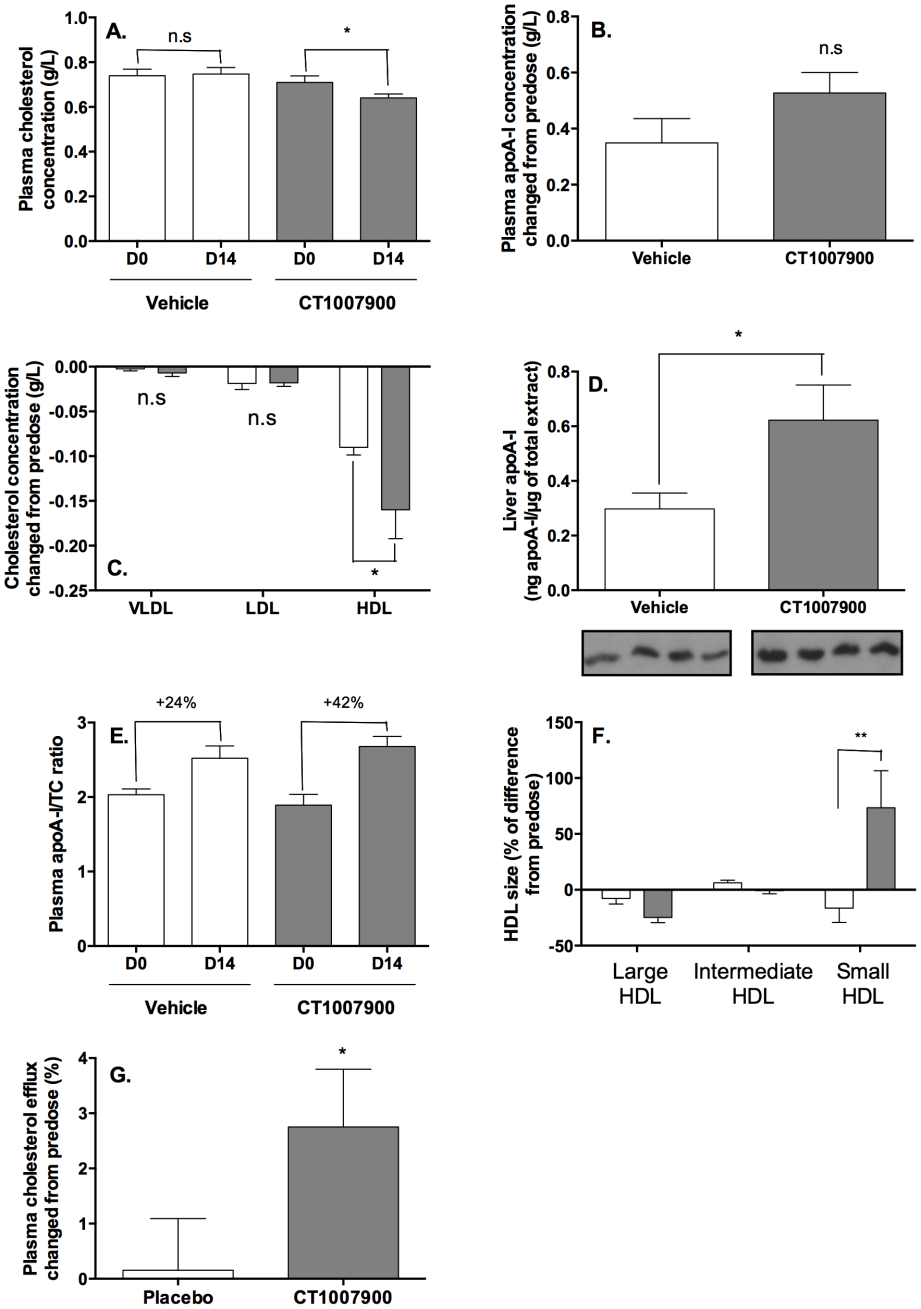
Repeated dosing of P2Y13R agonist decreases HDL-C. Plasma cholesterol (panel A), plasma apoA-I (panel B) and plasma lipoproteins (panel C) concentrations were determined after 2 weeks oral dose of P2Y13R agonist at 100 µg/kg (grey bars) and compared to vehicle treated animals (empty bars). Values for plasma apoA-I vary from 1.5 to 1.85 g/L. *p<0.05. Panel D, Concentration of liver apoA-I was determined by Western-blot quantification (n = 4 mouse/group) using imageJ software. 40 µg of total liver extract (vehicle or CT1007900 at 100 µg/kg) were separated on the same 12.5% SDS-PAGE and probed with goat anti-apoA-I antibody. *p<0.05. Panel E, the ratios of apoA-I/plasma cholesterol concentrations were determined and compared to their respective pre-dose values. Panel F, HDL from C57Bl/6J mouse plasma were separated according to the size of the different HDL particles using the Lipoprint system. The data were expressed as the percentage of difference for each HDL subpopulation set to the HDL population in the pre-dose animals. **p<0.01. Panel G, Determination of cholesterol efflux capacity of mouse plasma (1% v/v) using pre-loaded [^3^H]-cholesterol-oxLDL macrophages. The results are expressed as a percentage of cholesterol efflux corrected from pre-dose. Values for cholesterol efflux before correction from pre-dose vary from 12–15%. *p<0.05.

We then evaluated the effect of a 2-week oral treatment with P2Y13R agonist (CT1007900) on the lipid profile in C57Bl/6J mice. A significant decrease of cholesterol levels (Figure 2A) along with an increase in apoA-I levels (Figure 2B) was observed in the plasma of the treated animals at 100 µg/kg/day. This decrease of cholesterol level corresponds to only the HDL fraction without any effect on VLDL and LDL fractions (Figure 2C). The hepatic apoA-I protein (Figure 2D) expression was increased upon 2 weeks treatment.

These changes also lead to an increase in the number of small HDL particles in the plasma along with a significant increase of the apoA-I/TC ratio (Figure 2E). To further illustrate these findings, we measured size distribution of the HDL particles using Lipoprint technique in the plasma samples from treated animals (Figure 2F). There was a clear decrease in the number of large HDL particles while a significant increase was noted in the number of small HDL particles.

These small HDL particles are considered as more potent particles for the removal of cholesterol from atherosclerotic plaques by increasing efflux of cholesterol from the macrophages present in the lesions [25], [26] thus promoting regression of the atherosclerotic plaques [27]. The determination of cholesterol efflux from [^3^H]-cholesterol loaded J774 macrophages using the plasma of 2 weeks treated mice (Figure 2G) showed an increase efflux of cholesterol after treatment with the P2Y13R agonist, confirming that plasma HDL from treated animals are more efficient to efflux cholesterol from macrophages [25].

We further investigated the effect of the stimulation of the P2Y13R pathway by agonist on the atherosclerotic plaque lesions in apoE^−/−^ mice on a short term high-cholesterol diet (HCD). The P2Y13R agonist exhibited a similar effect on RCT (i.e., bile acid and cholesterol increase in liver) in apoE^−/−^ mice as observed in control C57Bl/6J mice previously (Figure S2).

**Figure 3:**
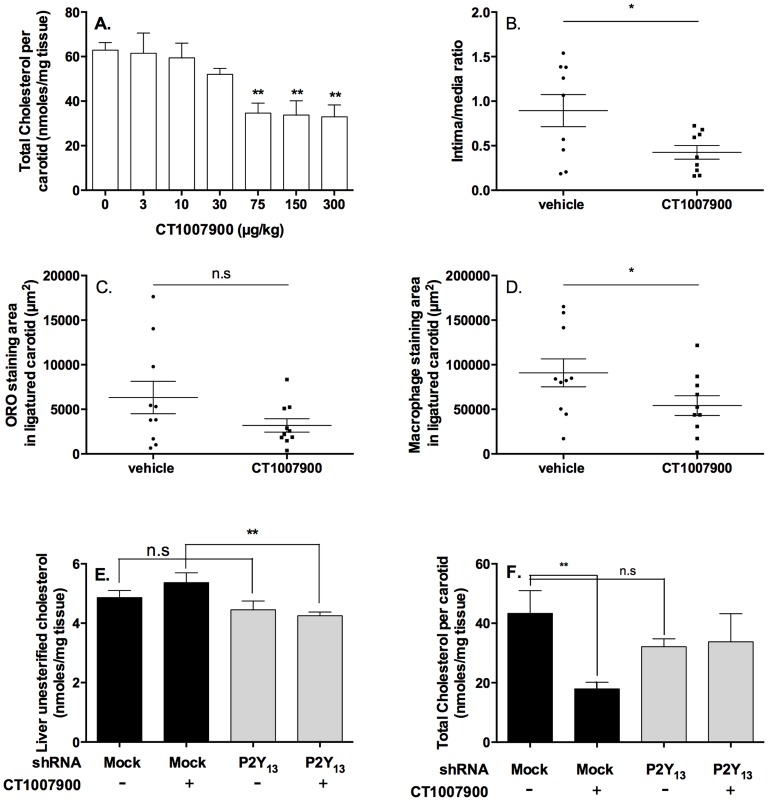
Effect of CT1007900 on atherosclerotic plaque progression in carotids of apoE^−/−^ mice. Pannel A. ApoE^−/−^ mice (n = 7) were ligatured on the upper part of the left carotids. On the day of the surgery the animals were placed on a HCD and given an oral gavage of vehicle or increasing doses of compound CT1007900. Ligatured carotids were lipid extracted in 2∶1 chloroform/methanol and the concentrations of total cholesterol were measured by HPLC. **p<0.01. Panel B, C and D: longitudinal sections of apoE^−/−^ mice ligated carotids were analyzed by hematoxylin eosin staining, Oil Red O staining and CD-68 antibody staining respectively. *p<0.05. Panel E: Liver unesterified cholesterol determination. **p<0.01. Panel F. ApoE^−/−^ mice (n = 10) were infected with 5×10^9^ adenoviral particles coding empty vector (mock) or vector encoding P2Y13R shRNA, 3 days before the ligation of the left carotid. On the day of surgery the animals were placed on a Western diet and also given oral gavage of vehicle or compound CT1007900 at 100 µg/kg, once a day for 2 weeks. Ligatured carotids were lipid extracted in 2∶1 chloroform/methanol. The concentrations in total cholesterol were measured by HPLC. **p<0.01.

We used a methodology described as “flow cessation model” in apoE^−/−^ mice [28]–[30], where the left carotid artery was ligatured to induce local inflammation before the addition of the diet. After 2 weeks of feeding with HCD, mice developed well-defined atherosclerotic lesions similar to the human pathology. Accelerated cholesterol plaque deposition was observed in the samples collected from the ligatured left carotid as we showed an increase in cholesterol content of about 2.3-fold as compared to the non-ligatured right carotid of the same animal (43.31+/−7.7 nmole of cholesterol/mg of tissue for left ligatured and 18.2+/−3.6 nmole of cholesterol/mg of tissue for right carotid). The concomitant dosing with the P2Y13R agonist for 2 weeks along with high-cholesterol diet showed a dose-dependent inhibition of the progression of the atherosclerosis lesions compared to control animals based on the lesion cholesterol content [31] (Figure 3A). The measurement of cholesterol content in carotids or aortas was chosen since the cholesterol is one of the main components of the atherosclerotic plaques and its decrease in plaque burden using for instance statin therapy had significant effect on the mortality and morbidity on the atherosclerosis [31]. Nevertheless, the quantification of plaque components by histo-pathological analysis of the treated carotids with hematoxylin-eosin, oil-red O staining and with CD68 antibody (specific of the macrophages), further confirmed this observation (Figure 3B to D). The staining clearly showed a decrease in lipid content of the carotids of the treated animals. In addition, to evaluate the P2Y13R specificity prior to carotid ligation and HCD, the mice were infected with adenovirus carrying either mock or specific shRNA targeted against the P2Y13R where a strong specific knock-down of hepatic P2Y13R was observed as compared to the sustained expression of hepatic P2Y1 receptors used as control (Figure S3A). As P2Y13R expression has also been shown to occur in brain and spleen of mouse [18], we ensured that those P2Y13R were not significantly affected by the adenovirus infection (Figure S3B and 3C). In addition, the cholesterol content of the liver of the mice was significantly decreased after silencing P2Y13R expression (Figure 3E). This observation confirmed as described above, that P2Y13R is involved in the HDL-cholesterol metabolism pathway. The concomitant dosing with CT1007900 (at 100 µg/kg/day) for 2 weeks along with high-cholesterol diet showed a decrease in the cholesterol content (60% decrease) of the ligatured carotids in mock adenovirus-treated mice (Figure 3F). By contrast, the effect of P2Y13R agonist on cholesterol content in carotids was completely abolished in mice treated with shRNA targeted against the P2Y13R (P2Y13R knocked-down), further supporting the specificity of the P2Y13R in this model (Figure 3F).

**Figure 4:**
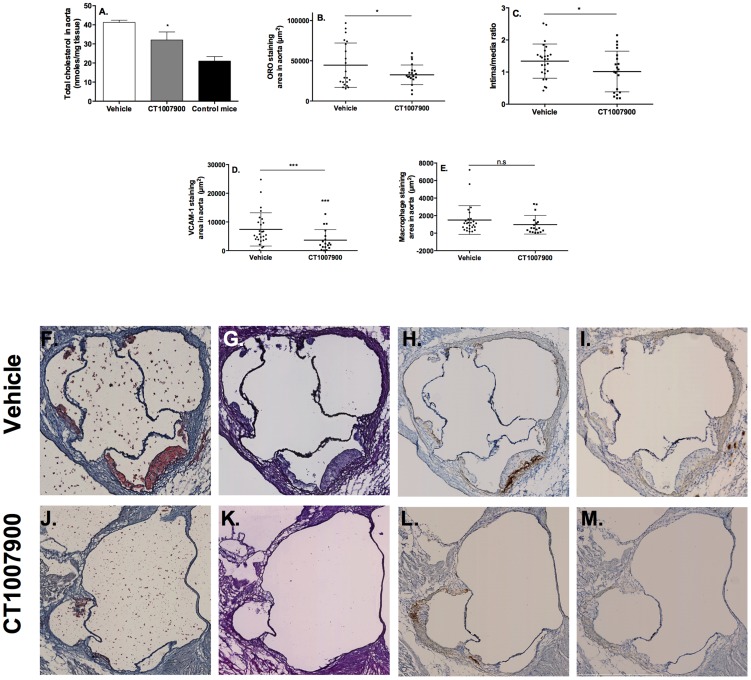
Effect of P2Y13R agonist on atherosclerotic plaque progression in aortas of apoE^−/−^ mice. ApoE^−/−^ mice (n = 20) were placed on a Western diet for 8 weeks and after one month of diet, they were dosed for 4 weeks with vehicle or 100 µg/kg of compound CT1007900. Aortas (n = 10) were lipid extracted in 2∶1 chloroform/methanol. Panel A, the concentrations in total cholesterol were measured by HPLC and GC/MS and compared to parallel apoE^−/−^ mice (n = 10) on normal Chow diet as a baseline Panel B, quantification of ORO staining area (n = 10). Panel C, quantification of the intima/media ratio (n = 10). Panel D, anti-VCAM1 antibody staining quantification area (n = 10). Panel E, F4/80 antibody staining quantification area (n = 10). Panel F to M, typical example of staining of aorta slides used for the quantifications. Panel F and J, ORO staining; panel G and K hematoxylin eosin staining; panel H and L, VCAM-1 antibody staining; panel I and M, macrophage staining of transversal sections of apoE^−/−^ mice aortas. *p<0.05, ***p<0.001.

The prevention of progression of the plaque in aorta was further evaluated in the cholesterol diet-fed apoE^−/−^ mice (Figure 4). Mice were placed on HCD for 8 weeks and after one month they were given CT1007900 at 100 µg/kg/day. The treated animals had significant decreases in cholesterol content (Figure 4A and 4B, 4F and 4J), plaque area (Figure 4C, 4G and 4K) and VCAM1 expression using specific antibody CD106 (Figure 4D, 4H and 4L) of the aortic wall; macrophage content was decreased (F4/80 antibody - Figure 4E, 4I and 4M) but did not reach statistically significance.

Altogether, these data suggest that P2Y13R is potentially involved in the prevention/regression of the atherosclerotic plaque as demonstrated using two different animal models, i.e. the apoE^−/−^ mice “flow cessation model” and the apoE^−/−^ mice HCD model.

## Discussion

The F1-ATPase/P2Y13R pathway was first described *in vitro* as key regulator of the HDL endocytosis by the hepatocytes. Further studies, in P2Y13R^−/−^ mice confirmed the implication of this pathway in HDL metabolism by impairing the reverse cholesterol transport in those animals.

Furthermore, it has been recently shown that infusion of Cangrelor, a compound already described *in vitro* as a partial agonist of P2Y13R, in mice for 3 days caused increase in the reverse cholesterol transport i.e. increase in the HDL-c clearance by the liver and subsequently increase of the bile acid secretion.

Here, using a novel P2Y13 agonist, CT1007900, we observed a decrease of the plasma HDL-cholesterol but more importantly a significant increase of the plasma small HDL-particles in mice rather than the large HDL-particles, following two weeks of treatment. This also led to an increase of the cholesterol efflux capacity of the treated plasma. Two mechanisms could explain this relative increase of the small HDL-particles. First, it was already described that the mature large HDL-particles were preferentially interacting with hepatocytes [32] as compared to smaller HDL-particles. Thus, using P2Y13R agonist would increase the clearance of the large HDL-particles. Second, we observed an increase in the liver of both apoA-I mRNA (data not shown) and protein expression of the treated animals. Even if we cannot exclude a direct effect of P2Y13R on apoA-I production as already described [33], it is likely that the uptake of large HDL particles by the liver also stimulates *de novo* synthesis of nascent HDL particles and enhanced the efflux capacity of the serum to eliminate cholesterol from atherosclerotic plaques.

P2Y13R was described in different tissues, mainly liver, brain and spleen [18]. To assess the tissue specificity of the P2Y13R agonist effects we knocked-down *in vivo* the P2Y13R expression in liver using sh-RNA. This completely abolished the effect of P2Y13R agonist on the atherosclerotic plaques. Furthermore, as part as the development of the agonists as potential therapeutics, their specificity was assessed in large numerous different targets. All together this allow us to conclude that the effect of P2Y13R agonists on atherosclerosis is not a consequence of other unknown mechanisms.

Even, if the data using adenovirus P2Y13R- shRNA infection in apoE^−/−^ mice, clearly emphasize the exclusive role of liver P2Y13R in the atherosclerosis inhibition we have observed, we cannot totally exclude the putative role of the P2Y13 agonist on brain and/or spleen P2Y13R in specific atherosclerotic disease such as re-infarction following a primary myocardial infarction as described recently. Indeed, Dutta et al [34] showed that after myocardial infarction or stroke, atherosclerotic apoE^−/−^ mice developed larger unstable atherosclerotic lesions with a more advanced morphology, which was associated with markedly increased monocyte recruitment. The authors demonstrated that myocardial infarction liberated hematopoietic stem and progenitor cells from bone marrow via sympathetic nervous system signalling (such as anxiety, pain, etc). Those progenitors then seeded the spleen yielding a sustained boost in monocyte production. We know today that the P2Y13R agonist did not seems to have an effect on the number of circulating monocytes after 4 weeks treatment of high-fat diet apoE^−/−^ mice (not shown). But thorough specific studies could be more demonstrative to assess the role of P2Y13R agonist on the re-infarction.

In conclusion, we demonstrate *in viv*o that P2Y13 receptors are key partners in the HDL metabolism and reverse cholesterol transport process, and thereby promoting the atherosclerosis protection. These data support a mechanism where the stimulation of the HDL uptake or endocytosis by the liver via P2Y13R pathway promotes cholesterol catabolism by the liver and secretion in the gallbladder. These results clearly demonstrate that improving functionality of HDL rather than the level of HDL could have a positive impact on the atherosclerotic pathology. These data also support that P2Y13R agonists could be highly useful pharmacological therapeutics for the treatment of complications due to atherosclerotic disease.

## Methods and Materials

### Animals

C57Bl/6J mice, 9 weeks old weighing approximately 18–20 g were purchased from Janvier (France). Animal housing and care were in compliance with the recommendations of Directive 86/609/EEC, and protocol approvals were obtained from institutional ethic committees.

### P2Y13 receptor agonist

CT1007900 (6-[1-(2-Dimethylaminopyrimidin-5-ylmethyl)-piperidin-4-yl]-2-morpholin-4-yl-pyrimidin-4-ol monohydrate) was custom synthesized by Alchem Laboratory, USA for Cerenis Therapeutics and the specific agonist activity of the molecule against P2Y13 were described in the submitted (Oniciu D.C. *et al* 2014 J. Med. Chem. Design, Synthesis and Biological Evaluation of Novel Non-Purinic P2Y13 Receptor Agonists as a Newly Proposed Treatment for the Cardiovascular Disease: Screening of a Small QSAR-Generated Library for P2Y13 Receptor Agonists as Prospective Candidates for Cardiovascular Therapies).

### Bile acid measurement

Bile acid concentration was determined with the Diazyme kit and validated by comparison with HPLC method [35]. Samples from mouse gallbladder were diluted 1∶5,000. For liver bile acid determination, 50 mg of tissue sample were extracted with PBS and analyzed with the Diazyme kit.

### Bile cholesterol and Plasma cholesterol

Bile cholesterol and Plasma cholesterol were determined according to the manufacturer protocol with enzymatic kit from Biolabo (France).

### Liver uptake

*In vivo* liver uptake experiment was adapted from previous works [36]. Mice were catheterized in the femoral vein at Physiogenex (Labège, France), 5 days before the initiation of experiment. Mice were fasted for 2 h prior to intravenous injection with CT1007900 at the dose of 10 nmole/kg, followed by an injection of isolated mouse HDL (15 µg/mouse) labeled with [^3^H]-cholesterol (10 µCi/mouse). Livers were washed with PBS and sampled at 2 h after the labeled-HDL injection. A slice of approximately 50 mg was homogenized in PBS and the radioactivity was determined by scintillation counting.

For feces analysis, mice were dosed with CT1007900 (100 µg/kg) followed by injection of mouse HDL (15 µg/mouse) labelled with [^3^H]-cholesterol (10 µCi/mouse). Feces were collected for 6 h, 24 h, 30 h and 48 h from individual housing cages and analyzed as previously described by Briand et al. [37].

### Plasma lipoproteins profiles

Lipoproteins profiles were measured by HPLC using a Sepharose 6 column and detected for total cholesterol with inline enzymatic detection [38] (Synelvia, Labège France).

### SELDI-TOF analysis

Mouse plasma (1∶50 dilution) were supplemented with apoA-I from homo-sapiens (1∶1 apoA-I_hs_∶apoA-I_mm_) as internal reference for determination of the mouse apoA-I concentration. ProteinChip Q10 was used as the matrix. The experiment was conducted by the protein profiling platform of IFR150, Toulouse, as previously described [39].

### Lipoprint profiles

Mouse plasma (25 µL) was analyzed with the Lipoprint HDL system (Quantimetrix corporation) according to the manufacturer protocol.

### Carotid and aorta cholesterol measurement

The samples were lipid extracted overnight at 4°C in CHCl_3_∶MeOH (2∶1) with addition of stigmasterol as internal standard. Samples were assayed for total cholesterol after saponification in methanolic KOH [40]. Carotid samples were analyzed by HPLC [41] and aortas by GC/MS (Synelvia, Labège, France). Both methods were previously validated and gave identical results.

### Cholesterol determination in apoE^−/−^ mouse liver

The analysis of cholesterol content in mouse liver was performed by “Le plateau MetaToul-LIPIDOMIQUE”, Toulouse, France. A pre-weighed amount of liver tissue was homogenized in 1 mL of methanol/5 mM EGTA (2∶1 v/v) with FAST-PREP (MP Biochemicals) and the equivalent of 0.5 mg of tissue was evaporated. Lipids were extracted according to Bligh and Dyer method in dichloromethane/methanol/water (2.5 ∶2.5 ∶2.1, v/v/v), in the presence of the internal standards : 3 µg of stigmasterol, 3 µg of cholesteryl heptadecanoate, 12 µg of glyceryl trinonadecanoate. Dichloromethane phase was evaporated to dryness, and dissolved in 20 µL of ethyl acetate. 1 µL of the lipid extract was analyzed by gas-liquid chromatography on a FOCUS Thermo Electron system using a Zebron-1 Phenomenex fused silica capillary columns (5 m×0,32 mm i.d, 0.50 µm film thickness).

### Animal protocol

The animal works were conducted according to the recommendations of European Directive 2010/63/UE, and protocol approvals were obtained from institutional ethic committees (Midi Pyrénées ethic committee, France). The animal works were conducted in animal care facility C.E.F, Prologue Biotech (agreement number A-31-254-01).

#### ApoE^−/−^ mice “flow cessation model”

Left carotid of apoE^−/−^ mice (9–10 week old) were ligatured and mice were placed on High Cholesterol diet (HCD). These mice were given CT1007900 once a day at 100 µg/kg (0.5% CMC, 0.2% Tween80) for 2 weeks.

#### ApoE^−/−^ “high cholesterol diet model”

Mice (9–10 week old) were placed for two month on HCD (0.2% cholesterol, 21% butter milk) and after one month of diet dosed with CT1007900 once a day at 100 µg/kg (0.5% CMC, 0.2% Tween80) for 4 weeks. The mice were sacrificed and the aortas were collected for biochemical and immunohistological characterizations. One set of aorta (n = 10) was detached at the base of the heart and placed in a glass tube with 3 mL of chloroform-methanol 2∶1 (v/v), and stigmasterol as the internal standard and then analyzed as described above. Another set of aortas (n = 10) was first extensively washed with PBS, then with 4% paraformaldehyde (500 µL) and finally with tissue tek OCT (500 µL). Next the aorta was embedded in OCT and frozen at −20°C. The blocks were cut at 20 µm to be near to the aortic root at the level of the 3 valves. From this moment, the sectioning thickness was set to 10 µm and 21 slides. Three step frozen sections (similar sections) of 10 µm separated one from the other were processed and stained with hematoxylin/eosin and analyzed by Histalim (Montpellier, France) after digitalization (Nanozoomer – Hamamatsu, Japan) using different software such as NDPview (Hamamatsu, Japan) and Visilog (Noesis, France). Briefly, the external edge of the aorta was manually determined, then algorithm segmented and measured the lumen, the intima-media area and the stained/labeled areas. Macrophages were detected using F4/80 primary rat monoclonal antibody (Abcam) and VCAM-1 was detected using rat anti mouse CD106 (AbDSerotec).

#### Adenovirus production and injection

The shRNA sequence targeting the mouse P2Y13, AGCTTTTCCAAAAAATCCTTTCCGACTCACACCTCTCTT GAAGGTGTGAGTCGGAAAGGATGGT, was cloned into pSUPER plasmid with H1 promoter. The adenovirus expressing the P2Y13 shRNA and the control adenovirus were produced by the platform of vectorology of IFR150, Toulouse – France, with a title of 0.8×10^11^ PFU/mL.

12 weeks old apoE^−/−^ mice were injected in the tail vein with 4×10^9^ adenoviral particles 3 days before left carotid ligature. These mice were fed with HCD and given CT1007900 once a day at 100 µg/kg (0.5% CMC, 0.2% Tween80) for 2 weeks.

#### Cholesterol efflux determination

Cholesterol efflux capacity was quantified in blood samples from mouse plasma collected before administration of the dose on day 0 and after 2 weeks of administration of CT1007900 as previously described [42]. Briefly, oxidized LDL (25 µg/mL), labelled with [^3^H]-cholesterol (2 µCi/mL; Perkin-Elmer), were added to J774 macrophages in culture for 24 h in 2.5% serum medium. [^3^H]-cholesterol release was measured after 6 h incubation of 1% (v/v) mouse plasma. All assays were performed in triplicate. Mouse plasma (calibrator) was included on each plate and the values were normalized by dividing plasma samples by this calibrator plasma to determine the normalized cholesterol efflux capacity of the samples [25].

#### QPCR analysis of liver samples from apoE^−/−^ mice

Fresh piece of mouse liver is homogenized in 1 ml TRIZOL and the mRNAs are extracted with the RiboPure kit (Ambion) and reverse transcribed with the High capacity RNA to cDNA kit (Applied Biosystems) according to the manufacturer protocols. Real time quantitative PCR for cyp7a1 is Taqman Mm00484152_m1 and for apoA-I is Taqman Mm00437569.m. The internal standard was HPRT-1. Each qPCR plate was normalized to their respective levels in liver sampled from mouse on normal chow diet (calibrator).

#### Statistical analysis

All statistical analysis were performed using T-test protocol with 95% confidence using Prism software (Graphpad).

## Supporting Information

Figure S1 Chemical structure of 6-[1-(2-Dimethylaminopyrimidin-5-ylmethyl)-piperidin-4-yl]-2-morpholin-4-yl-pyrimidin-4-ol monohydrate (CT1007900).
(TIFF)

Figure S2 Effect of P2Y13R agonist on liver bile acid and cholesterol concentrations in apoE^−/−^ mice. Left carotid of apoE^−/−^ mice (9–10 week old) were ligatured and mice were placed on Western diet. These mice were given CT1007900 once a day at 150 µg/kg (0.5% CMC, 0.2% Tween80) for 2 weeks. Bile acid (panel A) and cholesterol (panel B) contents of liver were evaluated using enzymatic kit for bile acids and HPLC for cholesterol. ** p<0.005, ***p<0.0005.
(TIFF)

Figure S3 P2Y13 silencing in apoE^−/−^ mice. ApoE^−/−^ mice were infected with 5×10^9^ adenoviral particles coding empty vector (mock) or vector encoding P2Y13R shRNA, After 2 weeks, samples from liver, brain and spleen were analysed by Western blot for P2Y13 content. Panel A, Western-blot of liver homogenates blotted with anti-P2Y13R or anti-P2Y1r antibodies. Panel B, Western-blot of brain and spleen homogenates blotted with anti-P2Y13R. Panel C, quantification of Western-blot (n = 6 for Mock shRNA and n = 13 for P2Y13R shRNA) from liver, brain and spleen homogenates probed with anti-P2Y13R. Quantification was performed with the use of imageJ software. *p<0.05.
(TIFF)
