# Supplementary figures and images for: P2Y13 Receptor Regulates HDL Metabolism and Atherosclerosis In Vivo
Source: PLoS One. 2014 Apr 25;9(4):e95807. doi: 10.1371/journal.pone.0095807 (PMC4000210; doi:10.1371/journal.pone.0095807)

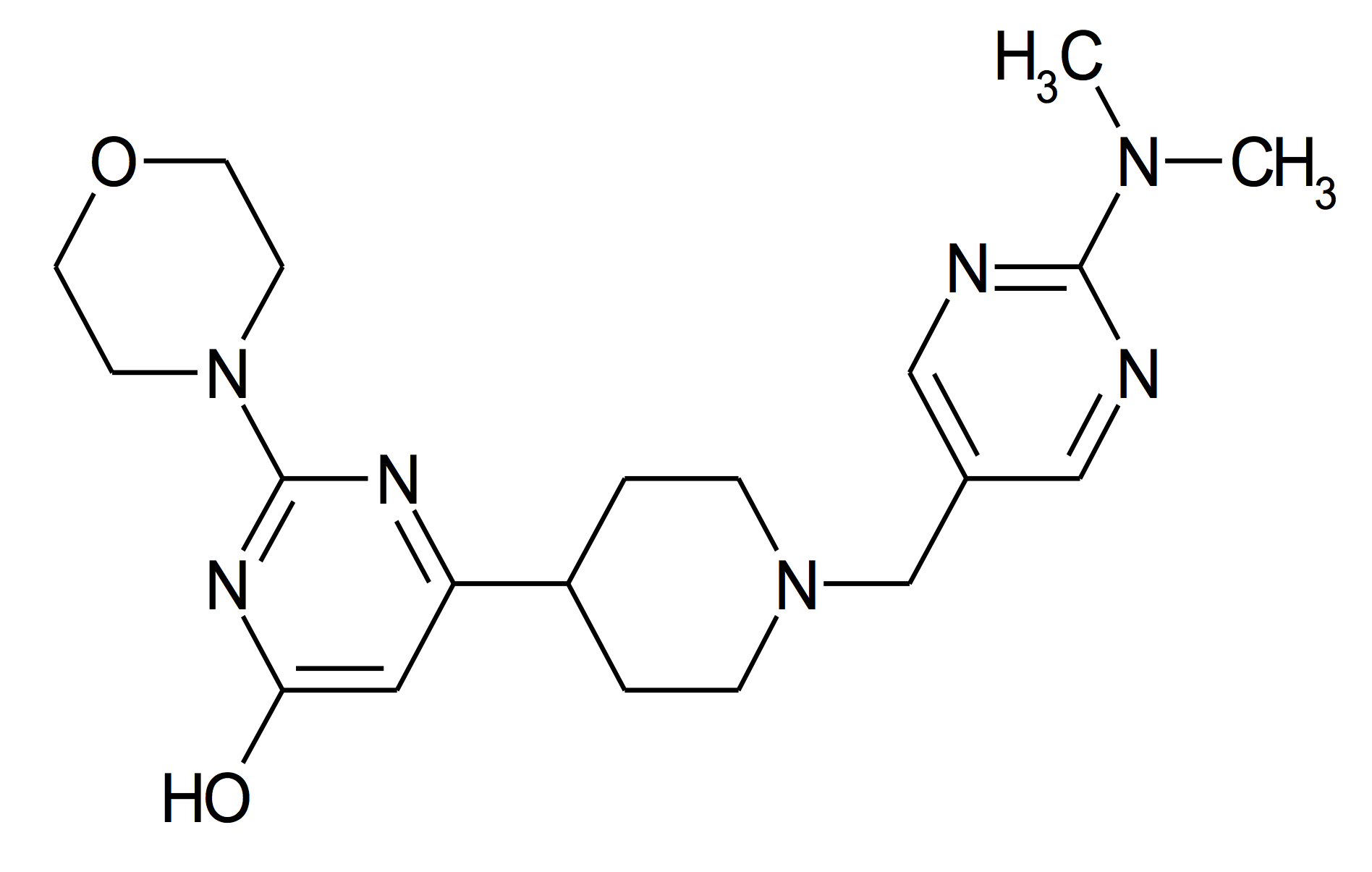

Supplement: Figure S1 — Chemical structure of 6-[1-(2-Dimethylaminopyrimidin-5-ylmethyl)-piperidin-4-yl]-2-morpholin-4-yl-pyrimidin-4-ol monohydrate (CT1007900). (TIFF) [file pone.0095807.s001.tiff]

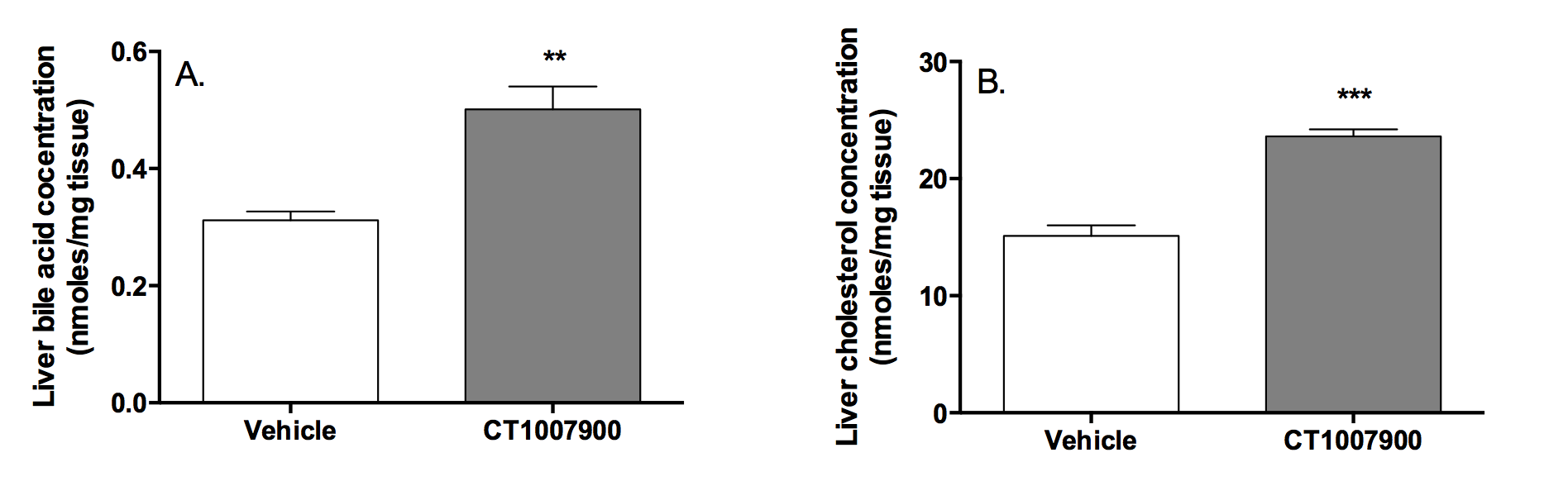

Supplement: Figure S2 — Effect of P2Y13R agonist on liver bile acid and cholesterol concentrations in apoE−/− mice. Left carotid of apoE−/− mice (9–10 week old) were ligatured and mice were placed on Western diet. These mice were given CT1007900 once a day at 150 µg/kg (0.5% CMC, 0.2% Tween80) for 2 weeks. Bile acid (panel A) and cholesterol (panel B) contents of liver were evaluated using enzymatic kit for bile acids and HPLC for cholesterol. ** p<0.005, ***p<0.0005. (TIFF) [file pone.0095807.s002.tiff]

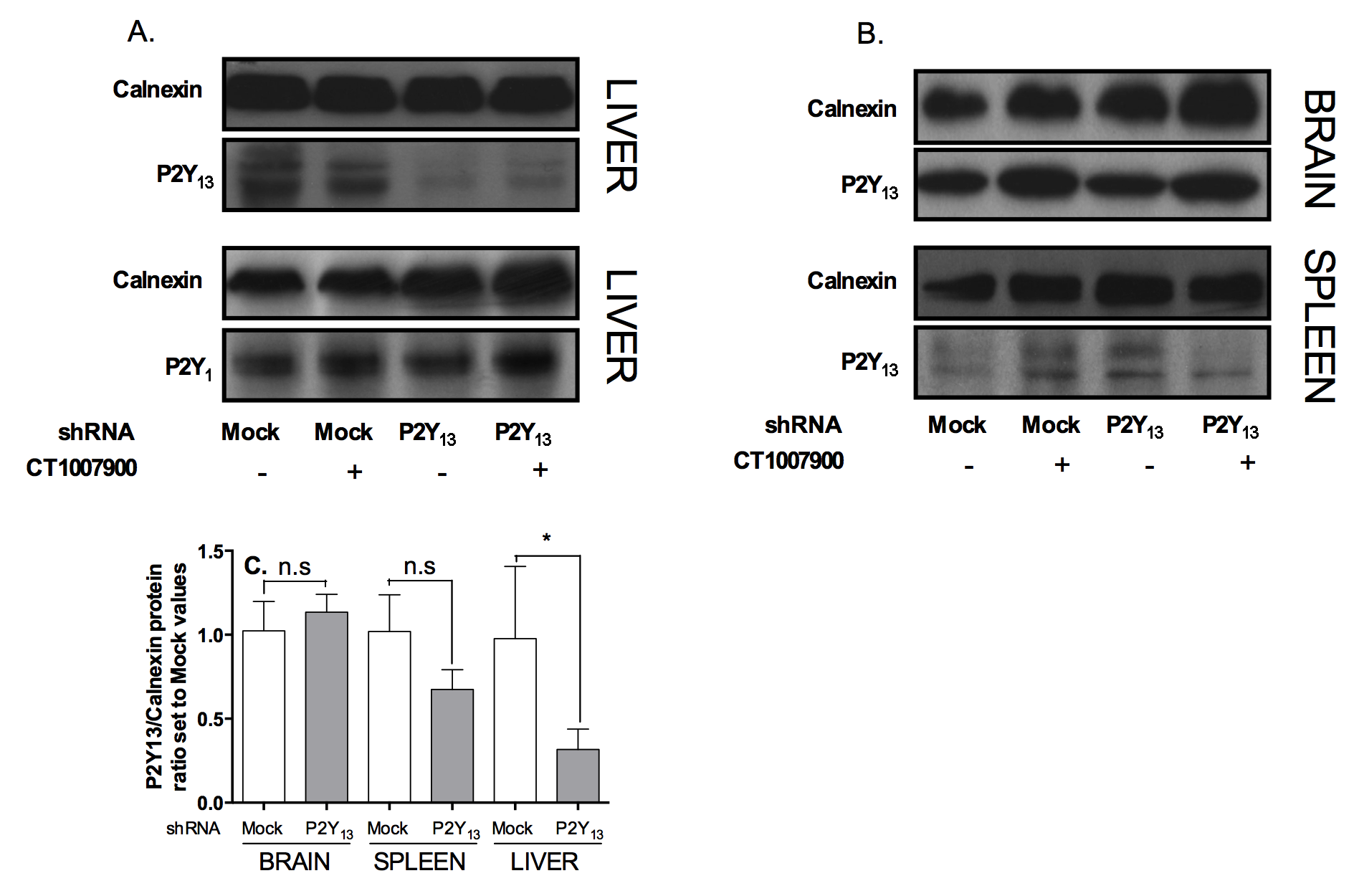

Supplement: Figure S3 — P2Y13 silencing in apoE−/− mice. ApoE−/− mice were infected with 5×10^9^ adenoviral particles coding empty vector (mock) or vector encoding P2Y13R shRNA, After 2 weeks, samples from liver, brain and spleen were analysed by Western blot for P2Y13 content. Panel A, Western-blot of liver homogenates blotted with anti-P2Y13R or anti-P2Y1r antibodies. Panel B, Western-blot of brain and spleen homogenates blotted with anti-P2Y13R. Panel C, quantification of Western-blot (n = 6 for Mock shRNA and n = 13 for P2Y13R shRNA) from liver, brain and spleen homogenates probed with anti-P2Y13R. Quantification was performed with the use of imageJ software. *p<0.05. (TIFF) [file pone.0095807.s003.tiff]
